# Step‐Associated Cu‐Ceria Interfaces Enhance Catalytic Activity and Selectivity

**DOI:** 10.1002/anie.5628406

**Published:** 2026-08-12

**Authors:** Shuxuan Feng, Shan Jiang, Chengyu Song, Chaochao Dun, Yang Deng, Hao Xu, Zihao Zhang, Xiong He, Temitayo Ojuetimi Ikuerowo, Mark R. Hoffmann, Jeffrey T. Miller, Yong Wang, Weixin Huang

**Affiliations:** ^1^ Department of Chemistry University of North Dakota Grand Forks North Dakota USA; ^2^ Davidson School of Chemical Engineering Purdue University West Lafayette Indiana USA; ^3^ The Molecular Foundry Lawrence Berkeley National Laboratory Berkeley California USA; ^4^ The Gene and Linda Voiland School of Chemical Engineering and Bioengineering Washington State University Pullman Washington USA; ^5^ Center for Renewable Carbon School of Natural Resources University of Tennessee Knoxville Tennessee USA; ^6^ Department of Energy Studies College of Engineering and Mines University of North Dakota Grand Forks North Dakota USA; ^7^ Institute for Integrated Catalysis Pacific Northwest National Laboratory Richland Washington USA

**Keywords:** CO_2_ hydrogenation, Cu nanoparticles, interface, location, methanol

## Abstract

Precise control of metal–support interactions is essential for understanding and steering catalytic processes. Here, we demonstrate that the surface location of metal nanoparticles (NPs) on oxide supports can strongly influence catalytic performance. Using atom‐trapped Cu catalysts as precursors enabled the formation of Cu NPs with distinct Cu–CeO_2_ interfacial environments compared with catalysts prepared by impregnation or colloidal methods. These resulting catalysts exhibit enhanced CO_2_ hydrogenation toward methanol formation by promoting formate activation. Enhanced reactivity arises from these Cu–CeO_2_ interfacial environments that render the methanol formation pathway thermodynamically more favorable, as revealed by theoretical calculations. These findings provide mechanistic insights into how NP anchoring environments influence catalytic activity and selectivity, offering a rational design strategy for oxide‐supported metal catalysts.

## Graphical abstract

The catalytic activity and selectivity of supported Cu nanoparticles depends on their interfacial environments, with Cu nanoparticles at step‐associated environments converting CO_2_ to methanol much faster than those on terrace facets, indicating that step sites create more active metal–oxide interfaces.

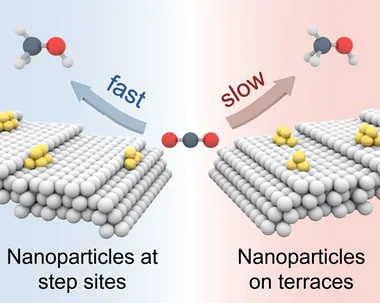

## 1 Introduction

Metal–support interaction is a fundamental factor that governs the catalytic behavior of both metal nanoparticles (NPs) and their oxide supports in heterogeneous catalysis [1, 2, 3, 4, 5]. Achieving precise control over these interactions has emerged as an effective approach to tailor catalytic activity and selectivity [6, 7, 8]. Oxide supports, with diverse surface structures, create a heterogeneous environment that profoundly influences the structural and electronic properties of anchored metal NPs [9, 10, 11]. This intrinsic heterogeneity governs the local chemical structure, which affects metal dispersion [12, 13, 14], and the formation of encapsulating oxide layers [15, 16, 17, 18], and ultimately determines reaction pathways, especially in reactions involving the active participation of lattice oxygen [19, 20, 21]. Despite growing recognition of metal–support interactions, fundamental questions remain: how metal NPs can be preferentially anchored at step sites, defect sites, or other undercoordinated regions on oxide surfaces, and whether the resulting distinct interfacial environments affect catalytic performance. Addressing these questions could guide the design of catalysts that leverage the unique properties of these sites in combination with metal NPs.

In this study, we demonstrate that the catalytic performance of copper (Cu) on cerium oxide (CeO_2_) is highly sensitive to the spatial location of Cu NPs on the support. It has been reported that atom trapping (AT) can anchor isolated metal atoms at undercoordinated CeO_2_(111) step environments on CeO_2_ [22, 23, 24, 25, 26, 27]. Building on this concept, we designed an atom‐trapping strategy to guide Cu NP formation toward these specific environments. Theoretical calculations suggest that atom‐trapped Cu species may provide favorable stabilization environments for subsequent Cu NP formation during reduction treatment. Cu NPs formed by this method exhibit a methanol space‐time yield (STY) about four times higher than that of catalysts prepared by wet impregnation or colloidal methods. The enhanced reactivity arises from distinct Cu–CeO_2_ interfacial environments, which facilitate formate‐to‐methanol conversion. These findings deepen our understanding of metal–support interactions and offer a general strategy for catalyst design through control of NP anchoring environments.

## 2 Results and Discussion

### 2.1 Preparation and Characterization of Cu/CeO_2_ and Cu_AT_CeO_2_ Samples

A Cu catalyst with approximately 1 wt% Cu loading was synthesized via the AT method [22, 28] and is referred to as Cu_AT_CeO_2_‐p, where “p” indicates the pristine, unreduced state. Following H_2_ reduction, which may promote the formation of Cu NPs, the catalyst is designated as Cu_AT_CeO_2_. For comparison, a reference catalyst was prepared by conventional impregnation, labeled Cu/CeO_2_‐p and Cu/CeO_2_ before and after H_2_ reduction.

Cu *K*‐edge X‐ray absorption near edge structure (XANES) spectra (Figure 1a) show that, relative to Cu and CuO references, the absorption edges of Cu_AT_CeO_2_‐p and Cu/CeO_2_‐p shift to higher energies, closely matching those of CuO. A Cu^2+^ pre‐edge peak is observed at ∼8.9775 keV, which is assigned to the dipole‐forbidden *1s* to *3d* transition. This indicates that the Cu species in both samples exhibit an average Cu valence similar to CuO. The corresponding Cu *K*‐edge extended X‐ray absorption fine structure (EXAFS) spectra (Figure 1b) display a single dominant peak attributed to the first‐shell Cu–O coordination. Notably, no spectral signatures attributable either to Cu–Cu coordination in metallic Cu foil or to the extended Cu–O–Cu network in CuO are observed. In both samples, there is a small higher shell peak, which fits well with a Cu–O–Ce scattering path, which strongly supports the atomic dispersion of Cu in both catalysts. The corresponding x‐ray absorption spectroscopy (XAS) fitting results are provided in Table S1.

**FIGURE 1:**
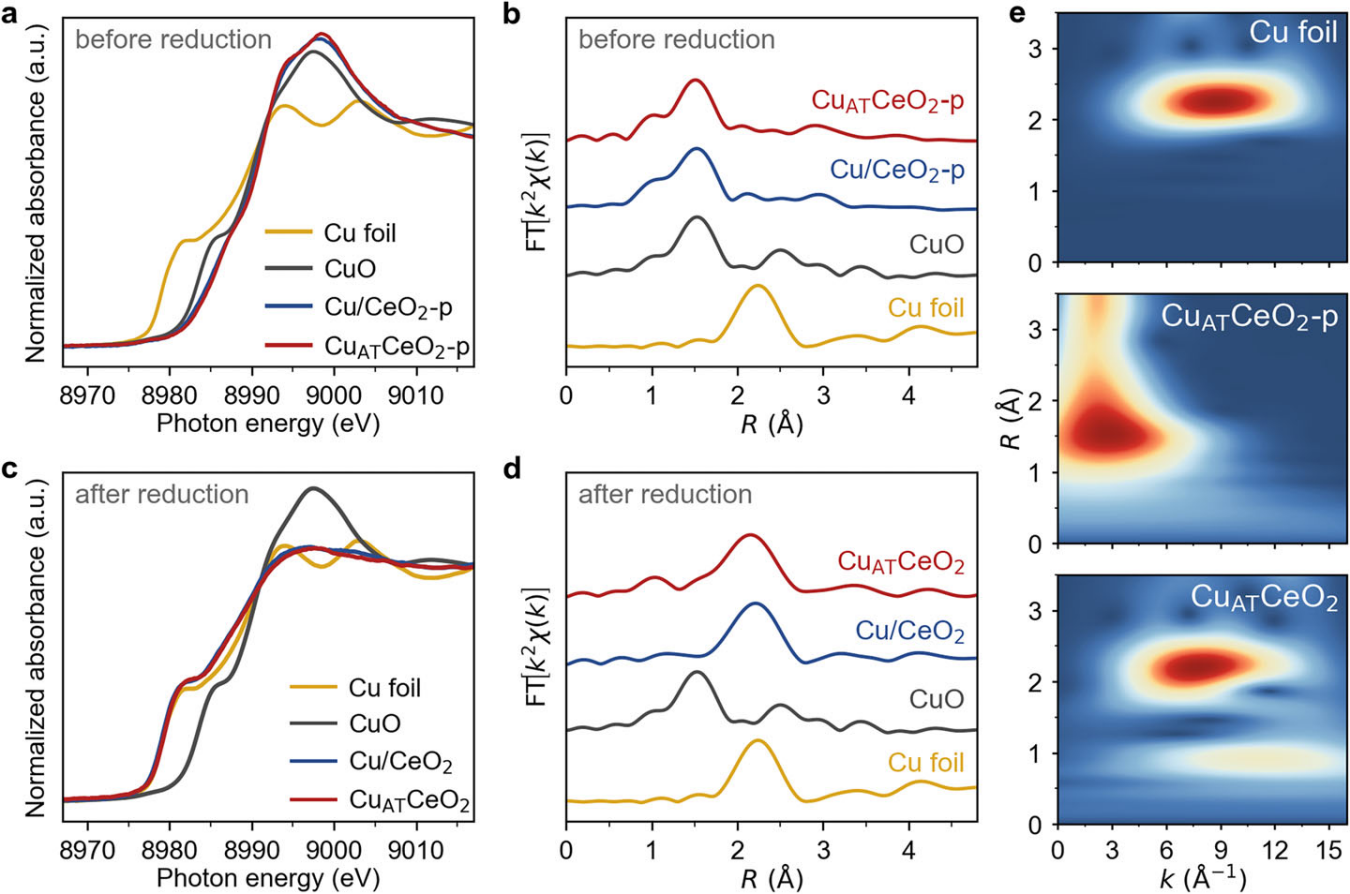
In situ catalyst characterization. (a) Cu *K*‐edge XANES spectra and (b) corresponding Fourier‐transform magnitudes of EXAFS spectra for pristine Cu/CeO_2_ and Cu_AT_CeO_2_, labeled as Cu/CeO_2_‐p and Cu_AT_CeO_2_‐p, respectively. (c) Cu *K*‐edge XANES spectra and (d) corresponding Fourier‐transform magnitudes of EXAFS spectra for Cu/CeO_2_ and Cu_AT_CeO_2_ after H_2_ reduction, labeled as Cu/CeO_2_ and Cu_AT_CeO_2_, respectively. (e) Wavelet transform analysis of the Cu *K*‐edge EXAFS spectra. Spectra of Cu foil and CuO references are shown for comparison. [Correction added on 13 August 2026, after first online publication: Figures 1b and 1d has been updated.]

To induce the formation of metallic NPs from single‐atom Cu species, the catalysts were subjected to a H_2_ reduction treatment. In situ XAS was then conducted immediately after H_2_ treatment, with the samples protected under Helium atmosphere to avoid changes caused by air exposure (Figure 1c,d). Under these experimental conditions, both catalysts exhibit a substantial shift in XANES edge energies to lower values, closely matching that of a metallic Cu foil reference. Notably, the XANES spectra show slight deviations from the Cu foil reference, consistent with known NP size effects that induce bond contraction and characteristic changes in XANES features, as previously observed in Pt and Au systems [28]. The corresponding EXAFS spectra show the emergence of a distinct Cu–Cu scattering feature at ∼2.50 Å, closely matching the Cu–Cu distance in metallic Cu, providing direct evidence for metallic Cu formation.

Notably, unlike the above in situ characterization, ex situ analyses did not confirm the extensive formation of metallic Cu NPs (Figure S1). Instead, XANES indicates that a significant fraction of the Cu centers remains in an oxidized state (Figure S1a). This can be inferred from the Cu *K*‐edge near‐edge structure, which exhibits spectral features characteristic of Cu(II) species. A dominant Cu–O scattering peak in the extended fine structure further supports the persistence of oxidized Cu environments (Figure S1b). These results indicate that metallic Cu formed during H_2_ treatment is readily reoxidized upon air exposure, causing the ex situ measurements to reflect an oxidized CuO‐like state rather than the intrinsic reduced structures present immediately after reduction.

### 2.2 CO_2_ Hydrogenation

The catalytic performance of CO_2_ hydrogenation to methanol was systematically evaluated for Cu/CeO_2_ and Cu_AT_CeO_2_ catalysts within a temperature range of 180°C–250°C and under a pressure of 3 MPa. As shown in Figure 2a, the Cu_AT_CeO_2_ catalyst exhibits notably higher methanol selectivity and space‐time yield (STY) than the Cu/CeO_2_ catalyst throughout the temperature range. At 200°C, the methanol STY over Cu_AT_CeO_2_ was nearly four times greater than that of Cu/CeO_2_. Moreover, Figure 2b illustrates methanol selectivity as a function of CO_2_ conversion for both catalysts, revealing that Cu_AT_CeO_2_ consistently maintains higher selectivity at comparable conversion levels. Kinetic analysis further demonstrated that the apparent activation energy for methanol formation over Cu_AT_CeO_2_ was 73.0 kJ mol^−1^, significantly lower than the 86.8 kJ mol^−1^ measured for Cu/CeO_2_ (Figure 2c). These differences in catalytic behavior suggest that Cu NPs may occupy distinct surface sites on CeO_2_, which critically influence methanol production. Beyond its enhanced activity, Cu_AT_CeO_2_ also displays excellent stability, retaining ∼90% of its methanol STY after approximately 100 h of continuous reaction (Figure 2d).

**FIGURE 2:**
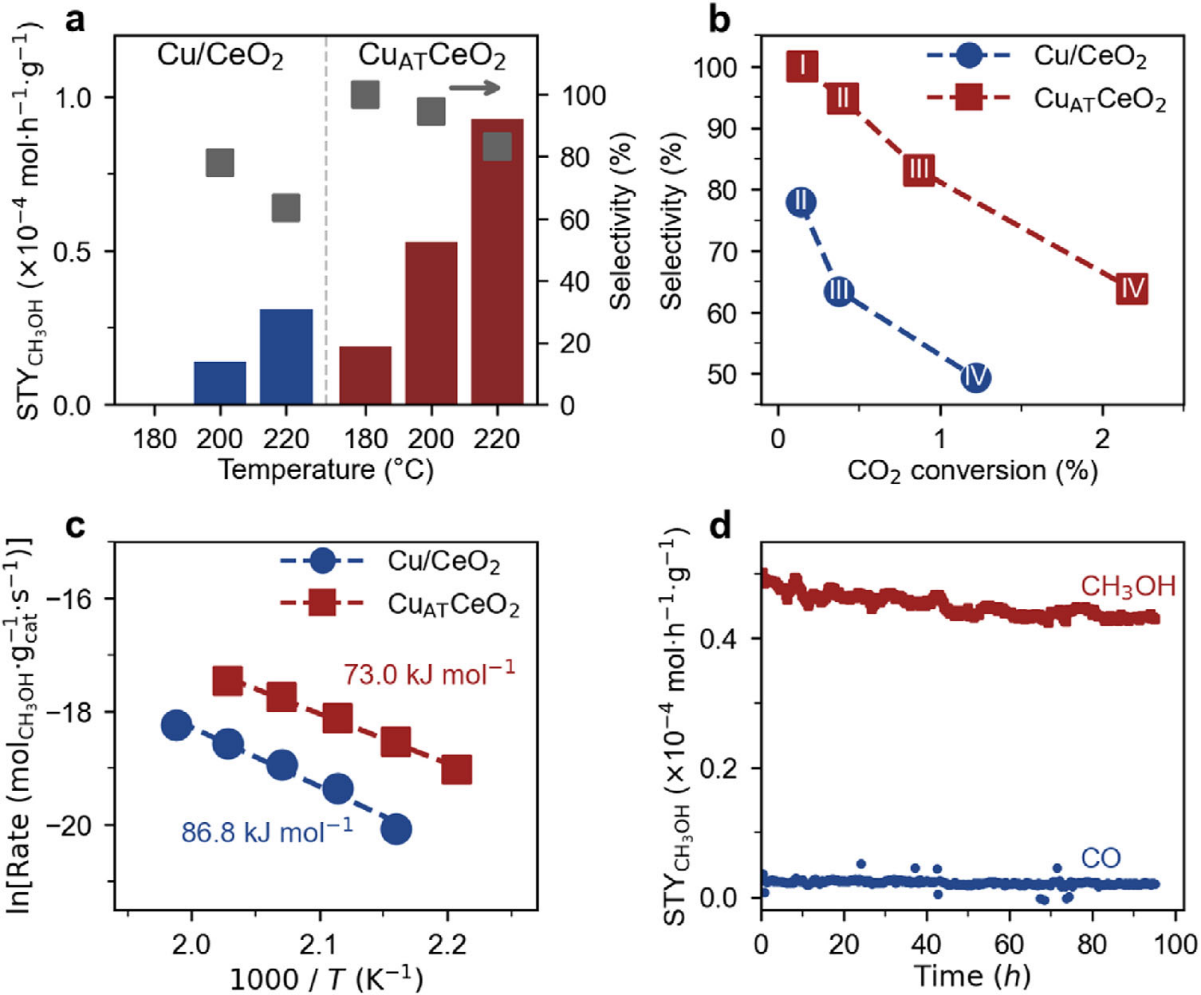
Catalytic evaluation. (a) Space‐time yield of methanol and methanol selectivity for Cu/CeO_2_ and Cu_AT_CeO_2_ measured between 180°C and 220°C. (b) Methanol selectivity as a function of CO_2_ conversion at different reaction temperatures over Cu/CeO_2_ and Cu_AT_CeO_2_; I to IV correspond to 180°C, 200°C, 220°C, and 250°C, respectively. (c) Arrhenius plots for CO_2_ hydrogenation to methanol over Cu/CeO_2_ and Cu_AT_CeO_2_. (d) Stability test of Cu_AT_CeO_2_ at 200°C. Reaction conditions: 0.5 g catalyst, 3 MPa total pressure, GHSV = 1320 mL·g_cat_^−1^·h^−1^, H_2_:CO_2_:Ar = 65.5:21.8:12.7 (volume %). All catalysts were pretreated under H_2_:Ar = 1:1 (30 mL min^−^^1^) at 300°C for 3 h.

### 2.3 Reaction Mechanisms

To elucidate the reaction intermediates and underlying mechanism, in situ diffuse reflectance infrared Fourier transform spectroscopy (DRIFTS) was conducted at 200°C and 3 MPa. Vibrational band assignments are detailed in Table S2. Upon exposure to a CO_2_–H_2_ mixture, both Cu_AT_CeO_2_ and Cu/CeO_2_ catalysts exhibited characteristic absorption bands assigned to formate species (HCOO*, 1330, 1380, 1550, 1587, and 2851 cm^−1^) and methoxy species (CH_3_O*, 2928 cm^−1^) [29, 30, 31, 32], as shown in Figures 3a,b, S2, and S3. The most notable difference lies in the significantly stronger methoxy signal on Cu_AT_CeO_2_. To capture the dynamic evolution of intermediates, the intensity ratio of methoxy to formate bands (CH_3_O*/HCOO*) was monitored over time (Figure 3c). This ratio consistently remained higher on Cu_AT_CeO_2_, indicating faster formate consumption coupled with accelerated methoxy formation. These differences in intermediate evolution reflect variations in the local environment of Cu NPs, which modulate the binding affinity and reactivity of surface species.

**FIGURE 3:**
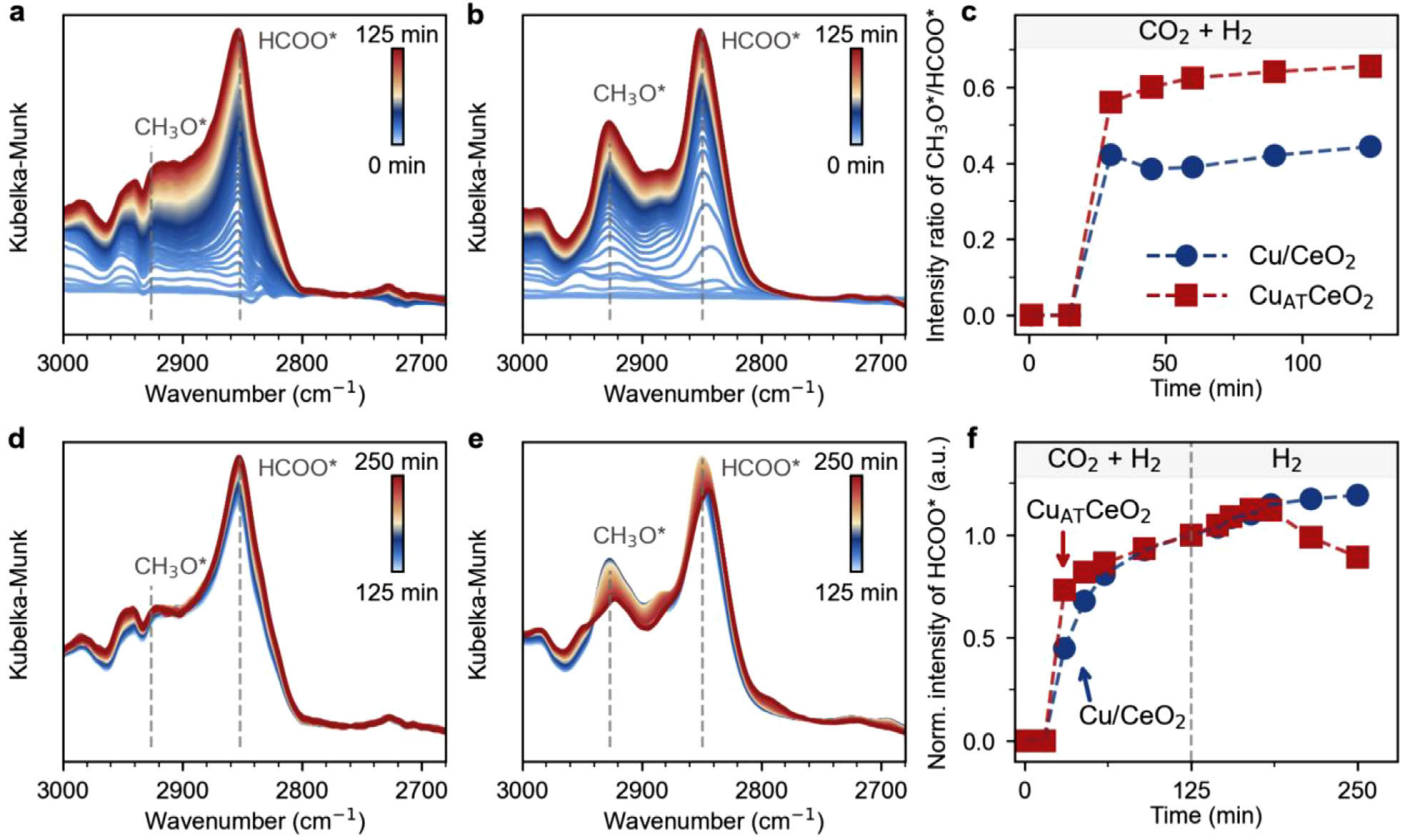
In situ characterizations of CO_2_ hydrogenation. In situ DRIFTS spectra of the CO_2_ hydrogenation reaction at 200°C and 3 MPa over (a) Cu/CeO_2_ and (b) Cu_AT_CeO_2_ catalysts. (c) Time evolution of the CH_3_O* to HCOO* peak intensity ratio derived from panels (a) and (b). In situ DRIFTS spectra of the reaction between H_2_ and surface‐adsorbed intermediates at 200°C and 3 MPa over (d) Cu/CeO_2_ and (e) Cu_AT_CeO_2_. (f) Normalized peak intensity of HCOO* as a function of reaction time (calculated from panels a, b, d, and e).

Following in situ DRIFTS measurements of CO_2_ hydrogenation, the catalysts were exposed to an H_2_/Ar atmosphere to assess the reactivity of surface‐adsorbed intermediates. As shown in Figure 3e, after the CO_2_ feed was stopped, the HCOO* signal on Cu_AT_CeO_2_ initially increased rather than decreased, which is attributed to residual CO_2_ remaining in the high‐pressure reactor and continuing to react on the surface. As this residual CO_2_ was gradually consumed, the intensities of both the HCOO* and CH_3_O* bands subsequently decreased, indicating their stepwise conversion from HCOO* to CH_3_O* and ultimately to methanol. In contrast, the HCOO* band on the Cu/CeO_2_ catalyst initially increased and then stabilized at a steady‐state intensity with no discernible decrease following CO_2_ removal, and the CH_3_O* species exhibited a similar trend but with a substantially smaller change in intensity (Figure 3d). These observations were further supported by the temporal intensity profiles of HCOO* and CH_3_O* (Figures 3f and S4). On Cu_AT_CeO_2_, the HCOO* signal (red curve) began to decline sharply after 170 min (Figure 3f), reflecting conversion to CH_3_O* intermediates. This step is recognized as the rate‐limiting process in CO_2_‐to‐methanol conversion [33, 34]. In contrast, on Cu/CeO_2_, the HCOO* signal (blue curve) showed no decline over the same period, indicating slower conversion to CH_3_O* under identical H_2_ exposure. A similar trend was observed for the CH_3_O* signal (Figure S4). On Cu_AT_CeO_2_, CH_3_O* decreases substantially over time, consistent with rapid conversion to methanol, whereas on Cu/CeO_2_, the signal declined only slightly, indicative of slower methanol formation. Overall, these results show that Cu_AT_CeO_2_ enables more efficient turnover of HCOO* and CH_3_O* intermediates, thereby accelerating the overall CO_2_‐to‐methanol conversion.

Several factors may explain the observed differences in catalytic activity between the CeO_2_‐supported Cu NP catalysts. Among these, surface oxygen vacancies are widely recognized as a key influence on CO_2_ adsorption and activation, as well as the stabilization of reaction intermediates. To examine whether variations in oxygen‐vacancy concentration contribute to the observed reactivity, we quantified the surface defect content using XPS, as shown in Figure 4a. Ce *3d* spectra show similar surface Ce^3+^ fractions for Cu/CeO_2_ and Cu_AT_CeO_2_ (23.6% vs. 25.5%), but Cu_AT_CeO_2_ exhibits an STY nearly four times higher. This disconnect indicates that the activity enhancement is not governed by differences in Ce^3+^ abundance. This finding is further supported by Raman spectroscopy. As shown in Figure 4b, both catalysts display a prominent *F*_2g_ vibrational mode characteristic of fluorite‐structured CeO_2_ at approximately 460 cm^−1^. Additionally, the broad Raman band centered around 600 cm^−1^, commonly associated with oxygen‐vacancy‐related defects in CeO_2_, shows nearly identical intensity in both samples. The consistent spectral features observed in XPS and Raman analyses indicate that the atomic trapping synthesis does not significantly alter the concentration of surface oxygen vacancies.

**FIGURE 4:**
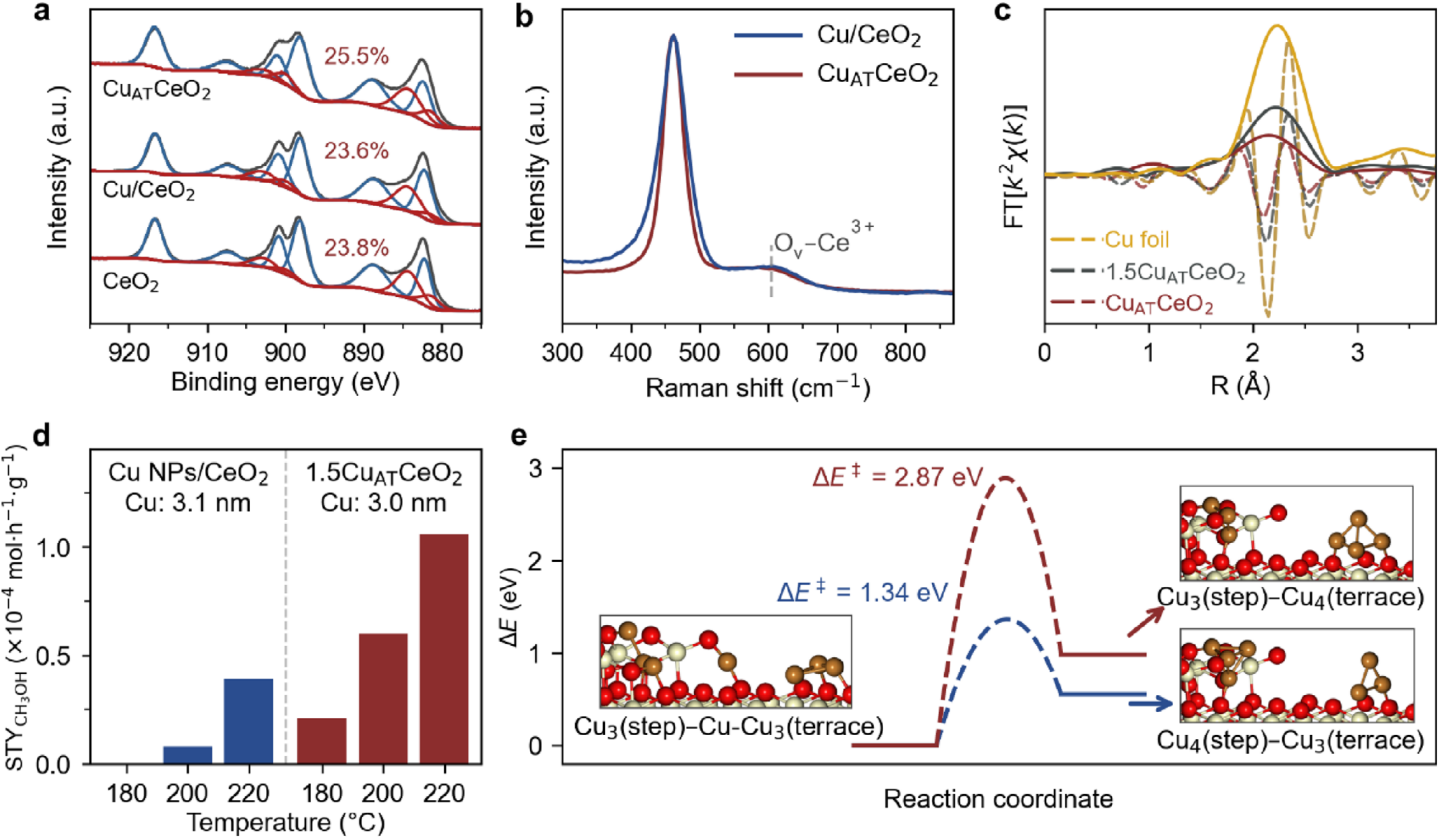
Investigation of factors contributing to catalytic activity enhancement. (a) Ce *3d* XPS spectra of Cu/CeO_2_ and Cu_AT_CeO_2_. (b) Normalized Raman spectra of Cu/CeO_2_ and Cu_AT_CeO_2_. (c) Fourier transforms (FT) of the *k* ^2^‐weighted Cu *K*‐edge EXAFS spectra for reduced 1.0 and 1.5 wt% Cu_AT_CeO_2_ catalysts compared with Cu foil. Solid lines show the FT magnitude and dotted lines the imaginary component. (d) Space‐time yield of methanol for Cu NPs/CeO_2_ and 1.5Cu_AT_CeO_2_ measured between 180°C and 220°C. Data are shown on an equivalent Cu basis to allow direct comparison among the samples. (e) Relative energy profiles for the migration of a Cu atom to Cu_3_ clusters at step and terrace sites.

The size of the metal NPs is a critical factor governing catalytic performance, as variations in particle size change both the fraction of reactive surface sites and the coordination environment of surface atoms, thereby modulating chemical reactivity [35, 36]. In this work, Cu NP sizes were estimated from coordination numbers derived by EXAFS analysis. As shown in Figure 4c, the Fourier transforms (FT) of the *k* ^2^‐weighted Cu *K*‐edge EXAFS spectra show clear distinctions between Cu_AT_CeO_2_ and bulk Cu foil. The supported catalysts display markedly lower FT magnitudes than metallic Cu, consistent with the presence of small Cu NPs. In addition, the imaginary components of the FT for both catalysts shift to lower R relative to the Cu foil, signifying contracted Cu‐Cu bond distances, a feature commonly associated with nanoscale Cu clusters [28]. A similar trend is evident in the *k*‐space spectra, where the oscillations show reduced amplitude and altered periodicity relative to Cu foil (Figure S5).

Quantitative EXAFS analysis further reveals differences in the local atomic environments of the reduced catalysts. As shown in Table S1, the Cu_AT_CeO_2_ catalyst shows an average Cu‐Cu coordination number (CN) of ∼4.5 at 2.48 Å, while the conventional Cu/CeO_2_ catalyst exhibits a higher CN of ∼6.7 at 2.52 Å. For comparison, bulk metallic Cu has a CN of 12 at a Cu–Cu distance of 2.55 Å, indicating that both catalysts contain much smaller Cu domains characterized by a high fraction of under‐coordinated surface atoms. These reduced coordination numbers and contracted Cu‐Cu bond distances confirm the formation of small metallic Cu NPs with a large proportion of surface atoms, in agreement with the size‐dependent spectral features observed in XANES. The Cu/CeO_2_ and Cu_AT_CeO_2_ catalysts exhibit average Cu–Cu coordination numbers of approximately 6.7 and 4.5, corresponding to mean particle diameters close to 2 and 1 nm, respectively. This pronounced size difference raises the question of whether the exceptionally small Cu NPs on Cu_AT_CeO_2_ drive its enhanced catalytic activity.

To evaluate this point, Cu_AT_CeO_2_ and conventional Cu/CeO_2_ catalysts with similar Cu NP sizes were examined. A Cu_AT_CeO_2_ sample with an increased Cu loading of 1.5 wt% was synthesized, denoted 1.5Cu_AT_CeO_2_, in which the higher loading is expected to favor the formation of larger Cu NPs. As shown in Figure 4c, the FT of the *k* ^2^‐weighted Cu *K*‐edge EXAFS spectra show that increasing the Cu loading from 1.0 to 1.5 wt% leads to higher Fourier transform magnitudes, indicating larger Cu NP sizes at higher metal loadings. EXAFS and XANES analyses confirmed that the electronic states and local coordination environments of Cu atoms in 1.5Cu_AT_CeO_2_ remained consistent with those of Cu_AT_CeO_2_, both before and after H_2_ reduction (Figure S6). In contrast, the 1.5Cu_AT_ CeO_2_ sample exhibited an increased average number of Cu–Cu bonds (7.4 at 2.52 Å), indicative of larger Cu NPs approximately 3 nm in size. Catalytic testing demonstrated that despite the larger NP size in 1.5Cu_AT_CeO_2_, its catalytic activity surpassed that of the smaller 2 nm Cu NPs in Cu/CeO_2_ (Figure S7). This observation challenges the prevailing assumption that smaller NPs intrinsically lead to higher catalytic activity.

To further probe the influence of particle size, Cu NPs with a uniform diameter of ∼3.1 nm were synthesized via a colloidal method (Figure S8) and subsequently deposited onto the CeO_2_ surface, yielding the Cu NPs/CeO_2_ catalyst. Despite having a comparable NP size to those in 1.5Cu_AT_CeO_2_ (∼3 nm), the Cu NPs/CeO_2_ catalyst exhibited significantly lower activity than 1.5Cu_AT_CeO_2_ under identical reaction conditions (Figures 4d and S9). This comparative analysis demonstrates that particle size does not account for the enhanced performance of Cu_AT_CeO_2_.

With the above factors ruled out, the enhanced catalytic reactivity in Cu_AT_CeO_2_ likely originates from alternative causes. In this context, the metal–support interface appears to be the most plausible contributor. Previous studies have shown that catalysts prepared via the AT method preferentially stabilize isolated metal species at undercoordinated CeO_2_(111) step‐associated environments [8, 22, 23, 24, 25, 26, 27, 37, 38, 39, 40, 41, 42]. In atom‐trapped Pt‐CeO_2_ systems, electron microscopy observations revealed atomically dispersed Pt species associated with ceria step‐edge structures [23, 39]. The formation of these structures was proposed to arise from interactions between mobile Pt–O species and undercoordinated Ce^3+^‐associated surface environments, leading to stabilization of interfacial Pt–O–Ce motifs during AT process [23]. Similar observations have also been reported for atom‐trapped Cu‐CeO_2_ systems, in which step‐associated trapping structures for isolated single‐atom Cu species were proposed based on the agreement between local coordination environments obtained from XAS and computational step‐defect structural models [42]. The polyhedral CeO_2_ support used in the present study was synthesized following procedures reported in previous atom‐trapping studies and is known to predominantly expose CeO_2_(111) facets together with surface step structures [22, 24, 26, 42]. By analogy with previous atom‐trapping studies, we propose that undercoordinated Ce^3+^‐associated environments may stabilize atomically dispersed Cu through Cu–O–Ce interactions and influence subsequent Cu NPs formation.

To test this hypothesis, DFT calculations were performed to compare the migration of a single Cu atom toward two types of Cu_3_ clusters: clusters anchored at step sites and clusters located on terrace sites. This comparison was designed to determine whether isolated Cu atoms preferentially migrate and aggregate at step‐bound Cu_3_ clusters rather than at terrace‐bound Cu_3_ clusters. As illustrated in Figure 4e, the computed migration energy barrier is lower for migration toward the step‐site Cu_3_ cluster (Δ*E* ^‡^ = 1.34 eV) than for migration toward the terrace‐site Cu_3_ cluster (Δ*E* ^‡^ = 2.87 eV), indicating that step sites facilitate Cu aggregation by reducing the kinetic barrier. Consistent with this trend, the step‐site pathway is also thermodynamically more favorable, with a smaller reaction energy (Δ*E* = 0.55 eV) compared with the terrace‐site pathway (Δ*E* = 0.98 eV). These results suggest that step sites serve as preferential trapping and nucleation centers, where a subset of Cu species is captured and initiates early‐stage cluster growth during reduction.

Atomic‐resolution HAADF‐STEM provides direct structural evidence for Cu‐rich nanoscale features associated with CeO_2_ surface steps in Cu_AT_CeO_2_ (Figure 5). The ceria particles predominantly expose (111) facets containing surface steps, highlighted by the red dashed lines in Figure 5a,b. Distinct low‐contrast nanoscale features are observed along the CeO_2_ surface‐step regions and can be distinguished from the surrounding ceria lattice. The corresponding EELS elemental maps show spatially coincident Cu signals, identifying these features as Cu‐rich regions. The projected thickness of the Cu‐rich features normal to the CeO_2_ surface is approximately 1 nm, consistent with the nanoscale Cu domains inferred from XAS. Similar step‐associated Cu‐rich features are consistently observed in additional Cu_AT_CeO_2_ images (Figure S10). In contrast, such Cu features are not observed in most examined regions of Cu/CeO_2_, with only sparse Cu species occasionally observed near CeO_2_ step edges (Figures S11 and S12). These observations support a preferential association of Cu species with step‐related environments in Cu_AT_CeO_2_ (Figure 6a). This structural assignment is consistent with the calculated energetics, which favor Cu aggregation at step‐associated sites (Figure 4e). The resulting step‐associated Cu–CeO_2_ interfaces therefore likely contribute substantially to the enhanced catalytic activity.

**FIGURE 5:**
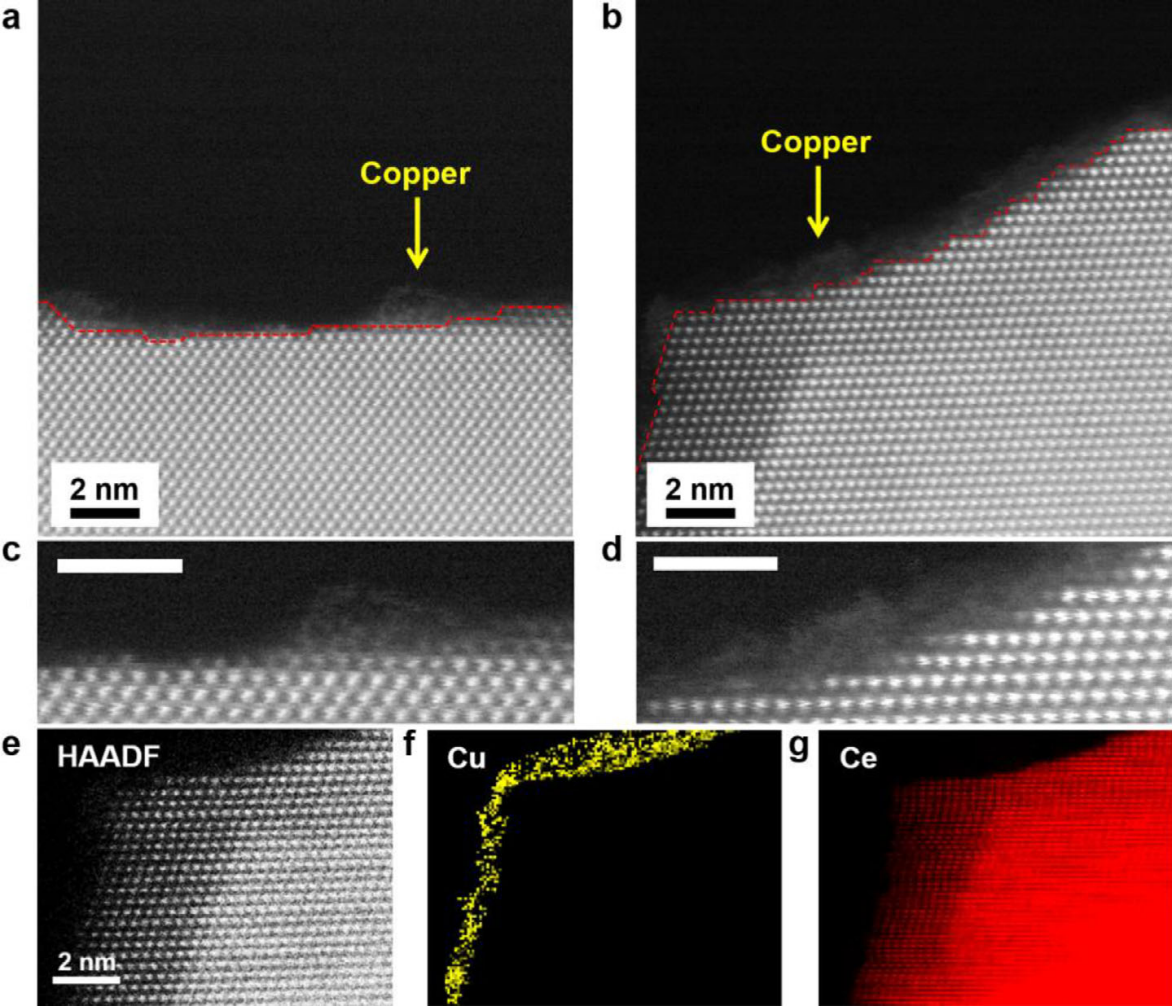
Atomic‐resolution STEM‐EELS characterization of Cu_AT_CeO_2_. (a, b) HAADF‐STEM images showing Cu‐rich nanoscale features associated with CeO_2_ surface steps. (c, d) Magnified images highlighting these features along the step regions. (e–g) HAADF‐STEM image and corresponding Cu and Ce EELS maps. Scale bars: 2 nm unless otherwise indicated.

**FIGURE 6:**
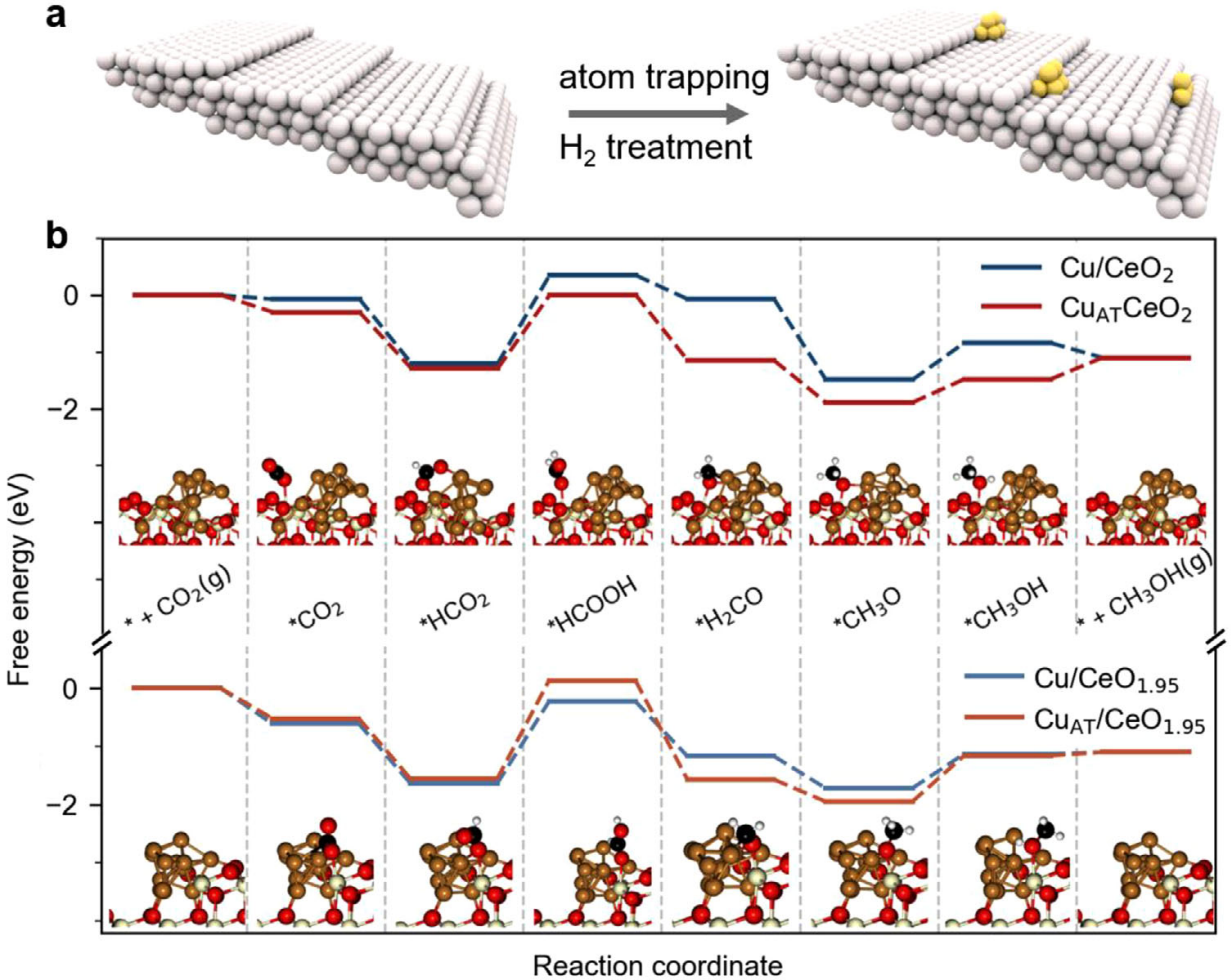
Validation of reaction pathways on the catalyst surface. (a) Proposed schematic illustration of Cu nanoparticle formation on Cu_AT_CeO_2_ after atom trapping followed by H_2_ reduction treatment. (b) Free energy diagrams for CO_2_ hydrogenation pathways on Cu/CeO_2_ and Cu_AT_CeO_2_. Cu, Ce, C, O, and H atoms are represented as brown, light yellow, black, red, and white spheres, respectively. Inset images depict the reaction pathway on Cu_AT_CeO_2_. CeO_2_ refers to the catalyst model without an oxygen vacancy. CeO_1.95_ indicates the introduction of an oxygen vacancy adjacent to the Cu site (O_v1_ in Figure S21), which is involved in interactions with formate intermediates during DFT calculations.

To further probe the local environments of Cu species in Cu_AT_CeO_2_ and Cu/CeO_2_, CO‐DRIFTS measurements were performed. As shown in Figure S13, although the CO‐DRIFTS spectra display similar CO adsorption peak positions for the two samples, their CO desorption behaviors differ markedly. After reaching saturated CO adsorption, the gas atmosphere was switched from CO to Ar, and the evolution of the CO–Cu bands was monitored over time. Following the Ar purge, the CO–Cu band intensity decreased substantially faster on Cu/CeO_2_ than on Cu_AT_CeO_2_. To quantitatively compare the CO desorption kinetics, the integrated CO–Cu peak intensities were plotted as a function of time, and the corresponding decay rates were extracted. As shown in Figure S13c,d, the CO–Cu band intensity decreases approximately three times more slowly on Cu_AT_CeO_2_ than on Cu/CeO_2_, indicating substantially stronger CO binding on Cu_AT_CeO_2_. These results provide evidence for distinct local Cu environments in the two samples.

To examine the local environments associated with Cu stabilization in Cu_AT_CeO_2_, DFT calculations of CO adsorption energies were performed on representative CeO_2_ surface models, including CeO_2_(111) terrace sites and low‐coordinated CeO_2_(111) step sites. The CeO_2_(111) terrace model was selected based on high‐resolution STEM analysis (Figures S14 and S15, Table S3), which revealed that CeO_2_(111) is the dominant exposed facet in both samples (∼65%–87%), while CeO_2_(110) and CeO_2_(100) are present in lower fractions. Because CeO_2_(111) is the dominant exposed surface in both samples, low‐coordinated CeO_2_(111) step environments were further considered as representative local coordination environments in the DFT calculations. The calculated Gibbs free energies for CO adsorption follow the order: Cu/CeO_2_(111) terrace < Cu/CeO_2_(111) step (Figure S16), indicating substantially stronger CO binding at low‐coordinated CeO_2_(111) step environments than at terrace environments. The markedly slower CO desorption observed experimentally for Cu_AT_CeO_2_ is consistent with stronger CO binding at step‐associated environments. Collectively, these experimental and computational results support that Cu species in Cu_AT_CeO_2_ are preferentially stabilized at low‐coordinated CeO_2_(111) step‐associated environments.

To elucidate the role of metal–support interfaces in CO_2_ hydrogenation, density functional theory (DFT) calculations were performed using representative Cu/CeO_2_ and Cu_AT_CeO_2_ interfacial models. The analysis focuses exclusively on the formate pathway, CO_2_* → HCOO* → HCOOH* → CH_2_O* → CH_3_O* → CH_3_OH*, which is widely regarded as the primary route for methanol synthesis during CO_2_ hydrogenation [43, 44]. Computed energy profiles and reaction pathways are presented in Figures 6b and S17–S20, with corresponding reaction energies summarized in Table S4. The Cu_13_ cluster model employed in this work represents a simplified structural model intended to capture the essential local Cu–CeO_2_ interfacial interactions. Similar Cu cluster models have been employed in prior DFT studies of supported Cu catalysts [45, 46, 47, 48]. Although the experimentally observed Cu NPs are larger (∼1–3 nm), the present calculations primarily focus on comparing the relative thermodynamic trends associated with different Cu–CeO_2_ interfacial environments. The interfacial structures considered here are representative computational models used to compare relative energetic trends.

Comparison of the catalysts indicates that Cu_AT_CeO_2_ provides a more favorable thermodynamic pathway for methanol formation. The free energy diagram suggests that the initial CO_2_ adsorption and activation steps are similar on both catalysts, as reflected by the small difference in CO_2_ adsorption energy and the modest stabilization of HCOO* on Cu_AT_CeO_2_. The major distinction instead appears in the subsequent transformation of HCOOH*‐derived intermediates to CH_3_OH*. On Cu_AT_CeO_2_, the reaction pathway following HCOOH* formation is thermodynamically more favorable, with lower free energies observed for the CH_2_O*, CH_3_O*, and CH_3_OH* intermediates. These results indicate that the primary role of Cu_AT_CeO_2_ is to facilitate the subsequent conversion of oxygenated intermediates and promote methanol formation through a more favorable reduction pathway.

To further evaluate whether the modeled Cu–CeO_2_ interfacial environments are representative of experimentally relevant structures, additional DFT calculations were performed on CeO_2_(110) and CeO_2_(100) surfaces under both stoichiometric and oxygen‐deficient conditions (Figures S22 and S23). Although the reaction energetics vary across different surface structures, similar energetic trends were observed, with low‐coordinated CeO_2_(111) steps exhibiting the most favorable thermodynamics for methanol formation among the examined models. CeO_2_(110) surfaces also exhibited competitive energetics under stoichiometric conditions; however, STEM analysis showed that the exposed fraction of CeO_2_(110) facets remained relatively low after H_2_ reduction in both samples (4%–9%). These results support the relevance of step‐associated Cu–CeO_2_ interfacial environments in Cu_AT_CeO_2_.

As discussed above, the two catalysts contained oxygen vacancies at comparable levels; however, oxygen vacancies may still modulate the reaction energetics and pathways. Our previous work demonstrated that oxygen vacancies on reducible oxides such as TiO_2_ and In_2_O_3_ promote CO_2_ activation and enhance methanol selectivity [21]. To extend this understanding to CeO_2_‐supported systems, Gibbs free‐energy profiles were further calculated using representative oxygen‐deficient Cu/CeO_2_ and Cu_AT_CeO_2_ interfacial models (Figures 6b, S24, and S25). As shown in Figure 6b, Introducing oxygen vacancies lowers the Gibbs free energies of several intermediates along the CO_2_‐to‐methanol pathway, including CO_2_*, HCOO*, CH_2_O*, and CH_3_O*, indicating that oxygen vacancies thermodynamically facilitate methanol formation through stabilization of adsorbed intermediates. Similar energetic trends are observed for both terrace‐associated and step‐associated Cu–CeO_2_ interfacial environments (Figures S24 and S25), although the step‐associated models generally remained more favorable for methanol formation. To further examine the electronic nature of oxygen vacancies, projected density of states (PDOS) analyses were performed on pristine and oxygen‐deficient Cu/CeO_2_ and Cu_AT_CeO_2_ models with adsorbed HCOOH* intermediates (Figures S26 and S27). The PDOS analyses reveal that oxygen‐vacancy formation shifts the Ce 4f states closer to the Fermi level, indicating increased Ce^3+^ character and enhanced surface reducibility. The step‐associated Cu_AT_CeO_2_ models also exhibit stronger energetic overlap between Ce 4f states and the O 2p states of adsorbed HCOOH* near the Fermi level, suggesting enhanced electronic coupling relevant to methanol formation. However, because the experimentally measured oxygen‐vacancy concentrations are comparable for Cu/CeO_2_ and Cu_AT_CeO_2_, differences in oxygen‐vacancy concentration are unlikely to represent the dominant origin of the enhanced methanol formation observed for Cu_AT_CeO_2_. Collectively, these results suggest that both oxygen vacancies and distinct Cu–CeO_2_ interfacial environments contribute to the reaction thermodynamics relevant to methanol formation.

## 3 Conclusion

In this work, we synthesized a series of Cu NPs supported on CeO_2_ and evaluated their performance in CO_2_ hydrogenation to methanol. Among these catalysts, Cu_AT_CeO_2_ exhibited markedly enhanced activity compared to conventional Cu/CeO_2_ and colloidal Cu NP counterparts, achieving higher methanol selectivity and STY. In situ DRIFTS measurements revealed that Cu_AT_CeO_2_ promoted the conversion of critical reaction intermediates, specifically facilitating the transformation of adsorbed formate (HCOO*) toward methoxy species (CH_3_O*). This conversion was less favorable on the Cu/CeO_2_ catalyst. Combined experimental and computational analyses attributed the catalytic differences to distinct Cu–CeO_2_ interfacial environments associated with atom‐trapping‐derived Cu species. DFT calculations suggest thermodynamically favorable Cu aggregation at step‐associated environments, while STEM‐EELS shows that Cu‐rich nanoscale features in Cu_AT_CeO_2_ are preferentially associated with CeO_2_ surface steps. These step‐site Cu–CeO_2_ interfaces offer a more favorable pathway for CO_2_‐to‐methanol conversion than terrace sites. Collectively, these findings provide mechanistic insight into how NP anchoring environments influence catalytic behavior in oxide‐supported metal catalysts.

## Author Contributions

**Shuxuan Feng**: data curation, writing – original draft, investigation, conceptualization, validation, formal analysis, visualization. **Shan Jiang**: investigation, data curation, formal analysis, resources, writing – review and editing, visualization. **Chengyu Song**: visualization, investigation, formal analysis. **Chaochao Dun**: investigation, formal analysis, resources. **Yang Deng**: data curation, software, formal analysis. **Hao Xu**: investigation, data curation. **Zihao Zhang**: formal analysis, conceptualization. **Xiong He**: investigation, validation, data curation. **Temitayo Ojuetimi Ikuerowo**: software, formal analysis. **Mark R. Hoffmann**: software, formal analysis. **Jeffrey T. Miller**: resources, data curation, formal analysis, investigation, conceptualization, writing – review and editing, funding acquisition. **Yong Wang**: conceptualization, project administration, supervision, data curation, formal analysis, writing – review and editing, funding acquisition. **Weixin Huang**: funding acquisition, conceptualization, investigation, methodology, software, formal analysis, data curation, supervision, resources, project administration, writing – review and editing, writing – original draft, visualization.

## Conflicts of Interest

The authors declare no conflicts of interest.

## Supporting information




**Supporting File**: anie74143‐sup‐0001‐SuppMat.docx.

## Data Availability

The data that support the findings of this study are available from the corresponding author upon reasonable request.
