# Supplementary material for: Step‐Associated Cu‐Ceria Interfaces Enhance Catalytic Activity and Selectivity
Source: Angew Chem Int Ed Engl. 2026 Aug 12;65(44):e5628406. doi: 10.1002/anie.5628406 (PMC13703893; doi:10.1002/anie.5628406)
Supplement: Supplementary file 1 [file ANIE-65-e5628406-s001.docx]

**Step-Associated Cu-Ceria Interfaces Enhance Catalytic Activity and Selectivity**

***Shuxuan Feng^1,^*** ^+^***, Shan Jiang^2,^*** ^+^***, Chengyu Song^3^, Chaochao Dun^3^, Yang Deng^1^, Hao Xu^4^, Zihao Zhang^5^, Xiong He^1^, Temitayo Ojuetimi Ikuerowo^6^, Mark R. Hoffmann^1^, Jeffrey T. Miller^2*^, Yong Wang^4, 7*^, Weixin Huang^1*^***

^1^Department of Chemistry, University of North Dakota, Grand Forks, ND 58202, USA

^2^Davidson School of Chemical Engineering, Purdue University, West Lafayette, IN 47907, USA

^3^The Molecular Foundry, Lawrence Berkeley National Laboratory, Berkeley, CA, 94720, USA

^4^The Gene and Linda Voiland School of Chemical Engineering and Bioengineering, Washington State University, Pullman, WA 99164, USA

^5^Center for Renewable Carbon, School of Natural Resources, University of Tennessee, Knoxville, TN, 37996 USA

^6^Department of Energy Studies, College of Engineering and Mines, University of North Dakota, Grand Forks, ND 58202, USA

^7^Institute for Integrated Catalysis, Pacific Northwest National Laboratory, Richland, WA 99354, USA

^+^These authors contributed equally.

*Corresponding authors: jeffrey-t-miller@purdue.edu (J. T. M.); wang42@wsu.edu (Y. W.); weixin.huang@und.edu (W. H.)

**EXPERIMENTAL SECTION**

**Chemicals and Materials**

Cerium (III) nitrate hexahydrate (Ce(NO_3_)_3_·6H_2_O, 99%), copper(II) nitrate hemi(pentahydrate) (Cu(NO_3_)_2_·2.5H_2_O, 99.99%), copper(I) acetate (CuAc), trioctylamine (TOA) and tetradecylphosphonic acid (TDPA) obtained from Sigma Aldrich. Isopropyl alcohol, ethanol and methanol were purchased from Fisher Scientific. H_2_ (99.999%), CO_2_ (99.9%) and Ar (99.9999%) (all gases from Airgas).

**Synthesis of Cu_AT_CeO_2_**

The 1 wt.% Cu_AT_CeO_2_ catalyst was synthesized using an atom-trapping approach. Initially, the CeO_2_ support was prepared through the thermal decomposition of Ce(NO_3_)_3_·6H_2_O at 350 °C in an air environment for a duration of 4 hours with a ramp rate of 1°C·min^-1^. Subsequently, a precise quantity of Cu(NO_3_)_2_·2.5H_2_O (1 wt.% based on the CeO_2_ foundation for Cu) was dissolved in 300 μL deionized water for the impregnation of 1 g CeO_2_ powders. A 300 μL solution was introduced to the CeO_2_ powder, followed by drying the impregnated sample at 80 °C in an air environment for 2 hours. Following this, the dried precursor underwent calcination in static air at 800 °C for 5 hours with a ramp rate of 5 °C·min^-1^. The resulting sample was denoted as Cu_AT_CeO_2_-p, where “p” represents the pristine catalyst prior to reduction. A second sample with a Cu loading of 1.5 wt.% was similarly prepared and denoted as 1.5Cu_AT_CeO_2_-p. Before catalytic measurements, the catalysts were reduced at 300 °C for 3 h under a flow of 50 vol% H_2_/Ar (30 mL min_−1_). The reduced catalysts are referred to as Cu_AT_CeO_2_ and 1.5Cu_AT_CeO_2_, respectively.

**Synthesis of Cu/CeO_2_ and Cu NPs/CeO_2_**

The Cu/CeO_2_ catalyst with the loading of 1 wt.% Cu was synthesized by wetness impregnation. A Cu(NO_3_)_2_·2.5H_2_O solution was introduced on CeO_2_ support. After impregnation, the samples underwent an overnight drying process at 80 °C in an air environment. Following this, the dried precursors underwent calcination in static air at 400 °C for 5 hours with a ramp rate of 5 °C·min^-1^. The resulting sample was denoted as Cu/CeO_2_-p, where “p” represents the pristine catalyst prior to reduction. Before catalytic measurements, the catalysts were reduced at 300 °C for 3 h under a flow of 50 vol% H_2_/Ar (30 mL min^−1^). The reduced catalysts are referred to as Cu/CeO_2_.

The uniform Cu Nanoparticles (NPs) were synthesized using a previously reported colloidal method.^[1]^ 10 mL TOA was added to a three-neck flask and heated at 130 °C for 30 minutes under N_2_ to remove water and dissolved O_2_. After cooling to room temperature, 0.1226 g of CuAc and 0.1392 g of TDPA were added. The solution was heated to 180 °C for 30 minutes and then to 270 °C for 30 minutes under N_2_. Subsequently, the heating mantle was removed, and the solution was cooled to 100 °C. Ethanol was added, and the solution mixture was centrifuged at 6000 rpm for 15 minutes. The Separated nanoparticles were washed with acetone and redispersed in hexane.

Thermogravimetric analysis (TGA) was performed to determine the metal concentration in the synthesized colloidal nanoparticle solution. An aluminum TGA pan was preheated on a hot plate at 100 °C, and 500 μL of the nanoparticle solution was added dropwise. The pan was subsequently heated in the TGA apparatus with a flow of 10 vol% O_2_ in Ar flow up to 500 °C and held until a stable mass was reached, confirming the complete removal of solvents and organics. The final mass of CuO was converted to Cu, and the resulting value was divided by the solution volume to calculate the metal concentration. To achieve a nominal final loading of 1 wt.% Cu, an appropriate volume of the metal nanoparticle solution was added to a dispersion of the support in hexanes under vigorous stirring. The mixture was left at room temperature overnight to complete deposition. The solid was recovered by centrifugation at 3000 rpm for 3 minutes, and the supernatant was discarded. The recovered solids were sieved to particles smaller than 180 μm. Before catalytic testing, all catalysts underwent ligand removal using a previously reported fast thermal annealing method.^[2]^ This involved placing the samples in a preheated 350 °C furnace for 7 minutes, followed by sieving below 180 μm to mitigate mass transfer limitations. The resultant material is labeled as Cu NPs/CeO_2_.

**Catalytic Measurements**

The catalytic tests were conducted in a fixed-bed flow reactor. Prior to the reactions, SiC materials were calcined at 800 °C for 5 hours. For each reaction, a mixture of 0.5 g of Cu catalyst and 4 g of SiC was prepared and loaded into a reactor with an internal diameter of 6.2 mm. The catalyst bed was positioned between two layers of quartz wool, which served to prevent displacement of the catalyst powder and to preheat the reactant gases. The reactor was heated using a furnace, and the temperature was monitored with a K-type thermocouple inserted into the catalytic bed. Before catalytic testing, each sample was pretreated at 300 °C for 3 hours under a flow of 50 vol% H_2_/Ar (30 mL·min^-1^). Following pretreatment, the reactor was cooled to room temperature and the reaction gas mixture (CO_2_/H_2_/Ar = 1:3:0.58, 66 mL min^-1^) was introduced into the reactor and pressurized to 3 MPa by a backpressure controller (Swagelok PGI-63C-PG1000-CAOX). The reactor was then heated to the desired temperature at a ramp rate of 20 °C·min^-1^ and maintained there for several hours. The total gas flow rate during the experiments was 11 mL·min^-1^, with a composition of CO_2_/H_2_/Ar in a molar ratio of 1:3:0.58. The reactor effluent was analyzed using an online mass spectrometer (Pfeiffer Vacuum GSD 350 OmnniStar) and a gas chromatograph (SRI 8610C Multiple Gas Analyzer 5). The connection between the reactor outlet and the gas chromatograph was heated to 130 °C to prevent product condensation (e.g., H_2_O, methanol). Methanol selectivity was defined as the ratio of methanol formed to the total products formed.

***In-situ* Diffuse Reflectance Infrared Fourier Transform Spectroscopy (DRIFTS)**

*In-situ* DRIFTS measurements were performed using a Jasco FT/IR 6X spectrometer equipped with a mercury-cadmium-telluride detector and a Harrick high-pressure reaction cell. For *in situ* CO_2_ hydrogenation DRIFTS measurements, the sample was initially treated in 50 vol% H_2_/Ar (30 mL·min^-1^) at 300 °C for 60 minutes, followed by cooling to 200 °C. The H_2_ valve was then closed, and the Ar flow was adjusted to 33 mL·min^-1^ and maintained for 30 minutes. Subsequently, the Ar flow rate was increased to 66 mL·min^-1^, and the pressure was set to 3 MPa using a backpressure valve. The Ar flow rate was then reduced to 33 mL·min^-1^ and maintained for another 30 minutes. Background spectra were collected under an Ar flow before introducing the reaction stream. The gas flow was then switched to a mixture containing CO_2_ (7.2 mL·min^-1^), H_2_ (21.6 mL·min^-1^), and Ar (4.2 mL·min^-1^) at a total flow rate of 33 mL·min^-1^ for CO_2_ hydrogenation, with spectra recorded for 125 minutes. Following this, the CO_2_ valve was closed, and the gas flow was switched to an H_2_/Ar mixture (21.6 mL·min^-1^ H_2_ and 11.4 mL·min^-1^ Ar) under identical temperature conditions, with spectra recorded for an additional 125 minutes. Throughout the measurements, DRIFTS spectra were collected every minute. The heating and cooling ramp rates were set at 20 °C·min^-1^ for all measurements. Spectra were acquired at a resolution of 4 cm^-1^ over 420 scans.

*In situ* CO-DRIFTS measurements were carried out using the same DRIFTS configuration described above. Prior to measurement, the sample was reduced in 50 vol% H_2_/Ar (30 mL min^−1^) at 300 °C for 60 min and subsequently cooled to 150 °C. The sample was purged under Ar at 20 mL min^-1^ at 150 °C for 30 min. The temperature then decreased to 50 °C under Ar, and a background spectrum was collected. Subsequently, 10% CO/He was introduced at a total flow rate of 20 mL min^-1^, and DRIFTS spectra were continuously recorded to monitor CO adsorption. After 10 min of CO exposure, the gas feed was switched back to Ar (20 mL min^-1^), and spectra were continuously recorded to follow CO desorption from the sample surface.

**Characterization Techniques**

Cu *K*-edge XAS spectra were conducted at the Brookhaven National Laboratory (BNL) National Synchrotron Light Source II (NSLS-II) 8-ID ISS beamline. The samples were ground into fine powder, loaded into the quartz in-situ cell, and tightened with Swagelok fittings. Two heating elements were placed near the *in situ* cells to control the temperature. The pristine samples were scanned first and then heated to 250 °C with H_2_ for 15 minutes, sequentially cooled down to room temperature to take the scans after H_2_ treatment. Two ion chambers with a Cu foil in between were placed behind the sample to calibrate energy.

WinXAS was used to fit the data. The XANES spectra were normalized with a linear baseline in the pre-edge region and a third-order polynomials in the post-edge region. At least 15 spectra were averaged to improve the data quality. The oxidation state was determined from the edge position for foil and pre-edge peak for Cu^2+^ ions. The EXAFS spectra were fitted in the k^2^ weighted Fourier transform of the EXAFS data from k = 2.5-10.8 Å^-1^. The EXAFS scattering paths were calculated using FEFF 6.0 software. The amplitude reduction factor (S_0_^2^) was calibrated as 0.8 by fitting the Cu foil reference (CN = 12 Cu–Cu at R = 2.55 Å). Debye-Waller factors (σ^2^) were refined from the first-shell scattering contribution.

The visible Raman spectra (532 nm) were collected on a Horiba LabRAM Soleil Raman with a Synapse Charge Coupled Device (CCD) camera. All Raman spectra were conducted at room temperature. The laser power was 0.88 mW and the exposure time was 10 s. HAADF HRSTEM was acquired on a FEI Titan 80-300 double aberration-corrected electron microscope, with an acceleration voltage of 300 keV and a probe convergent angle of 20 mrad. EELS mapping was acquired on a Gatan Continuum K3 with a collection angle of 80 mrad. X-ray photoelectron spectra (XPS) were obtained using a Thermo Scientific ESCALAB 250 Xi instrument with Al Kα radiation (1486.6 eV). Spectra deconvolution and quantification were performed using the CasaXPS program. All spectra were calibrated to their corresponding C *1s* peak and the U″′ component of the Ce *3d* core level, and binding energy values for all fitted and assigned spectral peaks are stated with an accuracy of 0.2 eV.

**Computational Details**

The crystallographic information files (CIFs) for CeO_2_ were obtained from the Materials Project Database, while the crystallographic data for Cu were sourced from the American Mineralogist Crystal Structure Database. Modeling and calculations were conducted using BURAI 1.3 and Quantum ESPRESSO.

In this study, the CeO_2_ (111) surface was selected due to its identification as a predominant exposed facet in TEM analyses. A step edge was constructed based on this surface. For the Cu/CeO_2_ system, the CeO_2_ (111) support supercell comprised eight CeO_2_ formula units, forming two O–Ce–O layers. The Cu_AT_CeO_2_ support supercell contained six CeO_2_ formula units, also forming two O–Ce–O layers. Oxygen vacancies on both catalyst support surfaces were modeled by selectively removing lattice oxygen atoms from the top layer. To simulate Cu nanoparticles on different supports, a classic Cu_13_ cluster model was employed.^[3–6]^

Calculations utilized the ultrasoft Vanderbilt pseudopotential method combined with the Perdew–Burke–Ernzerhof (PBE) exchange-correlation functional.^[7]^ A plane-wave cutoff energy of 400 eV was applied, and a vacuum thickness of 15 Å was maintained to avoid interactions between periodic images. The Brillouin zone was sampled at the Γ-point. Structural relaxation was performed using the pw.x module of Quantum ESPRESSO until the maximum force on any atom was below 0.03 eV/Å. The total energy (*E_DFT_*) of the optimized structures was recorded.

The Gibbs free energy change (Δ*G*) was calculated as:

Δ*G* = Δ*E_DFT_* + Δ(*ZPE* − *TS*)	(1)

where ZPE denotes the zero-point energy (eV), S is the entropy (eV/K), and the system temperature (*T*) was set to 473.15 K. Following previously established methods,^[8]^ the *ZPE* and entropy of adsorbates were determined from vibrational frequency calculations under the harmonic approximation, considering all 3N vibrational modes and excluding contributions from the slab. For these frequency calculations, the BLYP functional and def2-TZVP basis set were employed using ORCA 6.0.1,^[9]^ with DFT-D3 dispersion corrections applied.

Transition-state structures and energies were obtained from nudged elastic band (NEB) calculations after the energy profiles converged and became stable. A force convergence criterion of 0.1 eV Å^-1^ was applied, and the uncertainty in the calculated activation energies was limited to within 0.02 eV.

To construct representative oxygen-vacancy models, we first identified the Ce and lattice O atoms adjacent to the adsorbed intermediates. One or two nearby lattice O atoms were then selectively removed to generate single- and double-vacancy configurations, respectively. The vacancy positions were kept identical for all intermediates to ensure direct comparability. To further evaluate the vacancy-induced electronic interactions, we analyzed the PDOS of the Ce 4*f* states near the vacancy, the O 2*p* states associated with the corresponding lattice-oxygen sites in the vacancy-free reference structures. The PDOS calculations were performed using Quantum ESPRESSO through the BURAI 1.3 interface, with a 2 × 2 × 1 k-point mesh^[10]^ and Gaussian smearing of 0.136 eV. At least 896 electronic bands were included to ensure sufficient coverage of the unoccupied states. The PDOS was calculated from −50 to 50 eV relative to the Fermi level, using an energy interval of 0.01 eV and Gaussian broadening of 0.136 eV.

Figure S1. *Ex situ* XAS measurements. (**a**) Cu *K*-edge XANES spectra and (**b**) corresponding Fourier transform magnitudes of EXAFS spectra for Cu/CeO_2_ and Cu_AT_CeO_2_ after H_2_ reduction, labeled as Cu/CeO_2_ and Cu_AT_CeO_2_, respectively. Spectra of Cu and CuO references are also shown for comparison. Note that the samples were exposed to air before measurements.

Figure S2. *In situ* DRIFTS studies of CO_2_ hydrogenation at 200 °C and 3 MPa over Cu/CeO_2_, obtained by sequential exposure to (**a**) CO_2_ + H_2_ and (**b**) H_2_.

Figure S3. *In situ* DRIFTS studies of CO_2_ hydrogenation at 200 °C and 3 MPa over Cu_AT_CeO_2_, obtained by sequential exposure to (**a**) CO_2_ + H_2_ and (**b**) H_2_.

Figure S4. Normalized peak intensity of CH_3_O* as a function of reaction time (calculated from panels **a**, **b**, **d**, and **e** in Figure 3).

Figure S5. *k*^2^-weighted Cu *K*-edge Chi(k) spectra for Cu foil, reduced Cu_AT_CeO_2,_ and 1.5Cu_AT_CeO_2_ samples.

Figure S6. (**a**) Cu *K*-edge XANES spectra and (**b**) corresponding Fourier transform magnitudes of EXAFS spectra for 1.5 wt% Cu_AT_CeO_2_ before and after H_2_ treatment, labeled as 1.5Cu_AT_CeO_2_-p and 1.5Cu_AT_CeO_2_, respectively. Spectra of Cu and CuO references are also shown for comparison.

Figure S7. Space-time yield of methanol and methanol selectivity for Cu/CeO_2_ and 1.5Cu_AT_CeO_2_ measured between 180 and 220 °C.

Figure S8. Representative TEM images of Cu nanoparticles before support deposition and corresponding particle size distributions.

Figure S9. (**a**) Methanol selectivity as a function of CO_2_ conversion at different reaction temperatures over Cu/CeO_2_, 1.5Cu_AT_CeO_2_ and Cu NPs/CeO_2_; I to IV correspond to 180, 200, 220, and 250 °C, respectively. (**b**) Arrhenius plots for CO_2_ hydrogenation to methanol over Cu/CeO_2_, 1.5Cu_AT_CeO_2_ and Cu NPs/CeO_2_.

Figure S10. HAADF-STEM images of Cu_AT_CeO_2_. The square regions were magnified and shown in the upper-right insets with their contrast adjusted. The scale bar is 2 nm, unless marked in figures.

Figure S11. Atomic-resolution STEM-EELS characterization of Cu/CeO_2_. (**a,b**) HAADF-STEM images showing CeO_2_ surface steps. (**c,d**) Magnified views of the step regions. (**e–g**) HAADF-STEM image and corresponding Cu and Ce EELS elemental maps, showing only sparse Cu species on the CeO_2_ surface. Scale bars: 2 nm unless otherwise indicated.

Figure S12. HAADF-STEM images of Cu/CeO_2_. The square regions were magnified and shown in the upper-right insets with their contrast adjusted. The scale bar is 2 nm, unless marked in figures.

Figure S13. (**a,b**) Time dependent in situ CO-DRIFTS spectra of Cu/CeO_2_ and Cu_AT_CeO_2_ during CO desorption. (**c**) Normalized CO peak intensity on Cu/CeO_2_ and Cu_AT_CeO_2_ as a function of desorption time. (**d**) Linear fitting of the zoomed-in desorption region in panel c for Cu/CeO_2_ and Cu_AT_CeO_2_.

Figure S14. Atomic-resolution HAADF-STEM images of Cu_AT_CeO_2_. The dashed line indicates the region used for statistical analysis.

Note that the observed dark contrast features are attributed to internal voids within the CeO_2_ particles, which are commonly reported in CeO_2_ systems.^[11–14]^

Figure S15. Atomic-resolution HAADF-STEM images of Cu/CeO_2_. The dashed line indicates the region used for statistical analysis.

Figure S16. Calculated CO adsorption energies on Cu/CeO_2_(111) surfaces at different surface sites.

Figure S17. Reaction pathways of CO_2_ hydrogenation on Cu/CeO_2_ by DFT calculations. Color: Ce, yellow; O, red; Cu, brown; C, black; H, white. The catalyst model includes one oxygen vacancy.

Figure S18. Reaction pathways of CO_2_ hydrogenation on Cu_AT_CeO_2_ by DFT calculations. Color: Ce, yellow; O, red; Cu, brown; C, black; H, white. The catalyst model includes one oxygen vacancy.

Figure S19. Reaction pathways of CO_2_ hydrogenation on Cu/CeO_1.95_ by DFT calculations. Color: Ce, yellow; O, red; Cu, brown; C, black; H, white. The catalyst model includes one oxygen vacancy.

Figure S20. Reaction pathways of CO_2_ hydrogenation on Cu_AT_CeO_1.95_ by DFT calculations. Color: Ce, yellow; O, red; Cu, brown; C, black; H, white. The catalyst model includes one oxygen vacancy.

Figure S21. Optimized structure of the Cu/CeO_1.95_ and Cu_AT_CeO_1.95_ containing a single oxygen vacancy.

Figure S22. Gibbs free energy profiles for (**a**) CO_2_-to-CH_3_OH and (**b**) *HCOOH-to-CH_3_OH on Cu/CeO_2_ models with different exposed CeO_2_ surface structures under stoichiometric (0 O_V_) conditions.

Figure S23. Gibbs free energy profiles for (**a**) CO_2_-to-CH_3_OH and (**b**) *HCOOH-to-CH_3_OH on Cu/CeO_2_ models with different exposed CeO_2_ surface structures under oxygen-deficient (1 O_V_) conditions. The subscripts represent the O-to-Ce ratio in the DFT models, where one oxygen vacancy was introduced into each surface structure.

Figure S24. Gibbs free energy profiles for (**a**) CO_2_-to-CH_3_OH and (**b**) formate-to-CH_3_OH hydrogenation on Cu/CeO_2_(111)-terrace models with different oxygen vacancy concentrations.

Figure S25. Gibbs free energy profiles for (**a**) CO_2_-to-CH_3_OH and (**b**) formate-to-CH_3_OH hydrogenation on Cu/CeO_2_(111)-step models with different oxygen vacancy concentrations.

Figure S26. Structural models of oxygen vacancies and identification of key atoms for PDOS analysis on Cu/CeO_2_ and Cu_AT_CeO_2_ surfaces with adsorbed HCOOH.

Figure S27. Projected density of states (PDOS) analysis of oxygen-vacancy-induced electronic structure changes on Cu/CeO_2_ and Cu_AT_CeO_2_ surfaces. Panels (**a,b**) and (**c,d**) correspond to Cu/CeO_2_ and Cu_AT_CeO_2_, respectively.

Table S1. Quantitative EXAFS fitting parameters for the coordination environments of the pristine and H_2_-reduced samples.

| Sample | Cu^+2^ Pre-edge Energy, keV | Edge Energy, keV | Scatter | CN | R, Å | σ^2^  (× 10^3^) | E_o_, eV |
| --- | --- | --- | --- | --- | --- | --- | --- |
| Cu foil | - | 8.9789 | Cu-Cu | 12 | 2.55 | 7.5 | 5.6 |
| 1.5Cu_AT_CeO_2_-p | 8.9775 | 8.9860 | Cu-O | 3.8 | 1.94 | 5.0 | -0.1 |
|  |  |  | Cu-O-Ce | 1.9 | 3.26 | 10.0 | 4.5 |
| 1.5Cu_AT_CeO_2_ | - | 8.9792 | Cu-Cu | 7.4 | 2.52 | 10.0 | 2.8 |
| Cu_AT_CeO_2_-p | 8.9775 | 8.9860 | Cu-O | 3.8 | 1.94 | 5.0 | 0.9 |
|  |  |  | Cu-O-Ce | 2.3 | 3.32 | 10.0 | 4.4 |
| Cu_AT_CeO_2_ | - | 8.9793 | Cu-Cu | 4.5 | 2.48 | 10.0 | -0.4 |
| Cu/CeO_2_-p | 8.9775 | 8.9858 | Cu-O | 3.6 | 1.94 | 5.0 | 1.4 |
|  |  |  | Cu-O-Ce | 2.2 | 3.30 | 10.0 | 4.5 |
| Cu/CeO_2_ | - | 8.9792 | Cu-Cu | 6.7 | 2.52 | 10.0 | 2.5 |

Note: CN = coordination number; R = bond distance; σ^2^ = Debye-Waller factor; E_0_ referenced to Cu foil. Cu_AT_CeO_2_ and Cu/CeO_2_ contain 1 wt% Cu; 1.5Cu_AT_CeO_2_ contains 1.5 wt% Cu.

Table S2. Assignment of IR bands of surface species on the catalysts, based on literature references.

| **Surface species** | **Wavenumber (cm^-1^)** | **Assignment** |
| --- | --- | --- |
| Formate  (HCOO*) | 2851 | *ν*(CH) |
|  | 1587 | *ν_as_*(OCO) |
|  | 1380 | *δ*(CH) |
|  | 1550 | *ν_as_*(OCO) |
|  | 1330 | *ν_s_*(OCO) |
| Methoxy (CH_3_O*) | 2928 | *ν_as_*(CH) |
| Methanol (CH_3_OH) | 2949 | *ν_as_*(CH) |

Table S3. Surface facet distributions of Cu/CeO_2_ and Cu_AT_CeO_2_ determined from statistical analysis of TEM images.

| **Sample** | **Cu/CeO_2_** | **Cu_AT_CeO_2_** |
| --- | --- | --- |
| Number of regions | 17 | 12 |
| Fraction of {111} | 65% | 87% |
| Fraction of {110} | 9% | 4% |
| Fraction of {100} | 26% | 9% |

Table S4. Calculated reaction energies for elementary steps in the microkinetic model of the formate pathway on Cu/CeO_2_, Cu_AT_CeO_2_, Cu/CeO_1.95_ and Cu_AT_CeO_1.95_. CeO_1.95_ indicates the introduction of an oxygen vacancy adjacent to the Cu site (O_v1_ in Figure S21), which is involved in interactions with formate intermediates during DFT calculations.

| Reaction coordinate | Reaction equation | **Gibbs free energy (eV)** \| \| \|; Cu/CeO_2_ \| Cu_AT_CeO_2_ \| Cu/CeO_1.95_ \| Cu_AT_CeO_1.95_ | | | |
| --- | --- | --- | --- | --- | --- |
| 1 | CO_2_ (g) + * → CO_2_* | -0.07 | -0.30 | -0.61 | -0.52 |
| 2 | CO_2_* + H_2_ (g) → HCOO* + H* | -1.20 | -1.29 | -1.64 | -1.55 |
| 3 | HCOO* + H* → HCOOH* | 0.35 | 0.00 | -0.23 | 0.13 |
| 4 | HCOOH*+ H_2_ (g) → CH_2_O* + H* | -0.07 | -1.15 | -1.17 | -1.57 |
| 5 | CH_2_O* + H_2_ (g) → CH_3_O* + H_2_O (g) | -1.50 | -1.89 | -1.72 | -1.95 |
| 6 | CH_3_O* + H* → CH_3_OH* | -0.84 | -1.48 | -1.12 | -1.15 |
| 7 | CH_3_OH* → * + CH_3_OH (g) | -1.10 | -1.10 | -1.10 | -1.10 |

**Supplementary references**

[1]	L.-I. Hung, C.-K. Tsung, W. Huang, P. Yang, “Room-Temperature Formation of Hollow Cu_2_O Nanoparticles” *Advanced Materials* **2010**, *22*, 1910–1914.

[2]	M. Cargnello, C. Chen, B. T. Diroll, V. V. T. Doan-Nguyen, R. J. Gorte, C. B. Murray, “Efficient Removal of Organic Ligands from Supported Nanocrystals by Fast Thermal Annealing Enables Catalytic Studies on Well-Defined Active Phases” *J. Am. Chem. Soc.* **2015**, *137*, 6906–6911.

[3]	Z. Cui, S. Meng, Y. Yi, A. Jafarzadeh, S. Li, E. C. Neyts, Y. Hao, L. Li, X. Zhang, X. Wang, A. Bogaerts, “Plasma-Catalytic Methanol Synthesis from CO_2_ Hydrogenation over a Supported Cu Cluster Catalyst: Insights into the Reaction Mechanism” *ACS Catal.* **2022**, *12*, 1326–1337.

[4]	M. Bodiuzzaman, L. Fan, N. M Halappa, Y. Chen, Y. Yan, S. Thomas, R. K. Miao, Y. C. Xiao, J. Wu, R. Dorakhan, M. Abulikemu, O. El Tall, D. Sinton, H. N. Alshareef, O. F. Mohammed, E. H. Sargent, O. M. Bakr, “High-Nuclearity Copper Molecular Catalysts for Electrocatalytic CO-to-Acetate Conversion” *J. Am. Chem. Soc.* **2025**, *147*, 31713–31721.

[5]	V. K. Ocampo-Restrepo, L. G. Verga, J. L. F. Da Silva, “Ab Initio Study of the C–O Bond Dissociation in CO_2_ Reduction by Redox and Carboxyl Routes on 3d Transition Metal Systems” *J. Phys. Chem. C* **2021**, *125*, 26296–26306.

[6]	J. Wang, J. Cai, K.-X. Ren, L. Liu, S.-J. Zheng, Z.-Y. Wang, S.-Q. Zang, “Stepwise structural evolution toward robust carboranealkynyl-protected copper nanocluster catalysts for nitrate electroreduction” *Science Advances* **2024**, *10*, eadn7556.

[7]	K. Lejaeghere, G. Bihlmayer, T. Björkman, P. Blaha, S. Blügel, V. Blum, D. Caliste, I. E. Castelli, S. J. Clark, A. Dal Corso, S. de Gironcoli, T. Deutsch, J. K. Dewhurst, I. Di Marco, C. Draxl, M. Dułak, O. Eriksson, J. A. Flores-Livas, K. F. Garrity, L. Genovese, P. Giannozzi, M. Giantomassi, S. Goedecker, X. Gonze, O. Grånäs, E. K. U. Gross, A. Gulans, F. Gygi, D. R. Hamann, P. J. Hasnip, N. A. W. Holzwarth, D. Iuşan, D. B. Jochym, F. Jollet, D. Jones, G. Kresse, K. Koepernik, E. Küçükbenli, Y. O. Kvashnin, I. L. M. Locht, S. Lubeck, M. Marsman, N. Marzari, U. Nitzsche, L. Nordström, T. Ozaki, L. Paulatto, C. J. Pickard, W. Poelmans, M. I. J. Probert, K. Refson, M. Richter, G.-M. Rignanese, S. Saha, M. Scheffler, M. Schlipf, K. Schwarz, S. Sharma, F. Tavazza, P. Thunström, A. Tkatchenko, M. Torrent, D. Vanderbilt, M. J. van Setten, V. Van Speybroeck, J. M. Wills, J. R. Yates, G.-X. Zhang, S. Cottenier, “Reproducibility in density functional theory calculations of solids” *Science* **2016**, *351*, aad3000.

[8]	N. Han, Y. Wang, H. Yang, J. Deng, J. Wu, Y. Li, Y. Li, “Ultrathin bismuth nanosheets from in situ topotactic transformation for selective electrocatalytic CO_2_ reduction to formate” *Nat Commun* **2018**, *9*, 1320.

[9]	F. Neese, “The ORCA program system” *WIREs Computational Molecular Science* **2012**, *2*, 73–78.

[10] K. Zhang, G. Li, C. Zou, S. Chen, S. Li, Z.-K. Han, Y. Jiang, W. Yuan, H. Yang, M. V. Ganduglia-Pirovano, Y. Wang, “A CeO_2_ (100) surface reconstruction unveiled by in situ STEM and particle swarm optimization techniques” *Science Advances* **2024**, *10*, eadn7904.

[11] J. Jones, H. Xiong, A. T. DeLaRiva, E. J. Peterson, H. Pham, S. R. Challa, G. Qi, S. Oh, M. H. Wiebenga, X. I. Pereira Hernández, Y. Wang, A. K. Datye, “Thermally stable single-atom platinum-on-ceria catalysts via atom trapping” *Science* **2016**, *353*, 150–154.

[12] D. Jiang, Y. Yao, T. Li, G. Wan, X. I. Pereira-Hernández, Y. Lu, J. Tian, K. Khivantsev, M. H. Engelhard, C. Sun, C. E. García-Vargas, A. S. Hoffman, S. R. Bare, A. K. Datye, L. Hu, Y. Wang, “Tailoring the Local Environment of Platinum in Single-Atom Pt_1_/CeO_2_ Catalysts for Robust Low-Temperature CO Oxidation” *Angewandte Chemie International Edition* **2021**, *60*, 26054–26062.

[13] Y. Tang, Y. Wei, Z. Wang, S. Zhang, Y. Li, L. Nguyen, Y. Li, Y. Zhou, W. Shen, F. F. Tao, P. Hu, “Synergy of Single-Atom Ni_1_ and Ru_1_ Sites on CeO_2_ for Dry Reforming of CH_4_” *J. Am. Chem. Soc.* **2019**, *141*, 7283–7293.

[14] C. Brambila, J. Nutter, M. Molinari, D. C. Sayle, T. Sakthivel, S. Seal, G. Möbus, “Void space and secondary oriented attachment mechanisms in cerium oxide nanorods” *J Nanopart Res* **2022**, *24*, 227.
